# Autism, Joint Hypermobility-Related Disorders and Pain

**DOI:** 10.3389/fpsyt.2018.00656

**Published:** 2018-12-07

**Authors:** Carolina Baeza-Velasco, David Cohen, Claude Hamonet, Elodie Vlamynck, Lautaro Diaz, Cora Cravero, Emilie Cappe, Vincent Guinchat

**Affiliations:** ^1^Laboratory of Psychopathology and Health Processes, University Paris Descartes-Sorbonne Paris Cité, Boulogne-Billancourt, France; ^2^INSERM U1061, Neuropsychiatry: Epidemiological and Clinical Research, Department of Emergency Psychiatry and Acute Care, CHU Montpellier, Montpellier, France; ^3^Department of Child and Adolescent Psychiatry, Reference Center for Rare Psychiatric Diseases, APHP, Groupe Hospitalier Pitié-Salpêtrière, Université Sorbonne, Paris, France; ^4^Institut des Systèmes Intelligents et Robotiques, CNRS UMR 7222, Université Sorbonne, Paris, France; ^5^University Paris-Est Créteil, Créteil, France; ^6^UMR-S 1075 INSERM/UNICAEN, Caen, France

**Keywords:** autism, joint hypermobility, Ehlers-Danlos syndrome, pain, genetic disorders, comorbidity

## Abstract

Autism Spectrum Disorder (ASD) and Joint Hypermobility-Related Disorders are blanket terms for two etiologically and clinically heterogeneous groups of pathologies that usually appears in childhood. These conditions are seen by different medical fields, such as psychiatry in the case of ASD, and musculoskeletal disciplines and genetics in the case of hypermobility-related disorders. Thus, a link between them is rarely established in clinical setting, despite a scarce but growing body of research suggesting that both conditions co-occur more often than expected by chance. Hypermobility is a frequent sign of hereditary disorders of connective tissue (e.g., Ehlers-Danlos syndromes, Marfan syndrome), in which the main characteristic is the multisystem fragility that prone to proprioceptive and motor coordination dysfunction and hence to trauma and chronic pain. Considering the high probability that pain remains disregarded and untreated in people with ASD due to communication and methodological difficulties, increasing awareness about the interconnection between ASD and hypermobility-related disorders is relevant, since it may help identify those ASD patients susceptible to chronic pain.

## Introduction

Autism Spectrum Disorders (ASD) is a blanket term for an etiologically and clinically heterogeneous group of neurodevelopmental disorders commencing in early childhood. The core characteristics of ASD are impairments in communication, social interaction, and restricted repetitive and stereotyped behaviors (1). The prevalence of ASD is estimated to be around 1% (2). Most of the cases are “idiopathic” (i.e., unknown cause), and approximately 10% of cases are considered as “secondary autism” since these coincide with a genetic syndrome with identified etiology (3).

The burden of these long-lasting and disabling conditions is enhanced by an important degree of comorbidity, which is higher than observed in general pediatric population (4). In autistic adults, it has been reported that only 16% present good physical health (5). Unfortunately, somatic comorbidities in ASD have not been well-addressed in research settings (6). There are, however, more and more genetic syndromes that are identified as associated with autism (7).

Concerning painful conditions specifically, these are highly prevalent in the general population but remain under-diagnosed and under-researched in ASD (8). This is due to communication and methodological difficulties, but also to the late awareness among the medical community of the ability of autistic people to feel and express pain (8, 9). Indeed, this was questioned for decades. Nevertheless, nowadays it is well-accepted that individuals with autism do experience and express pain but in an atypical way (e.g., altered sensory thresholds, hypo- and hyper-responsiveness including behavioral problems) (2, 9, 10).

Although the magnitude of the co-occurrence between chronic pain and neurodevelopmental disorders such as ASD remains unknown (11), some data suggest that chronic pain is frequent among the ASD population. Bursch et al. (12) reported that more than 20% of pediatric patients in a pain clinic in US presented with ASD traits. In addition, potential pathological sources of pain such as neurologic disorders (seizures and epilepsy) and gastrointestinal problems are known to be frequent in ASD (13, 14). In addition, people with ASD are particularly exposed to pain due to aberrant behaviors such as self-injuries, aggressions, and agitation (13). Conversely, these disruptive behaviors as well as acute behavioral crisis can be manifestations of an underlying pain-associated pathology (15).

Thus, as Clarke [(2), p. 1] stated, “failure to recognize ASD as a common cause of pain can lead to late diagnosis, inappropriate treatment, distress, and further disability.” In this sense, it is necessary to disseminate knowledge concerning somatic pain conditions associated with ASD. This will help overcome the challenge of recognizing pain-related suffering which could worsen ASD symptoms and the general state of those affected.

Joint hypermobility (JH) refers to an exaggerated increase in the range of a given joint's mobility. This somatic trait is more frequent in infancy, decreases with age, and is more common in women than in men (16). When hypermobility is polyarticular (five joints or more), it is thought to be a congenital and hereditary trait caused by an alteration of collagen synthesis (17). Its prevalence has been estimated between 10–30% in males and 20–40% in females (16).

To have JH implies increased flexibility but also a propensity for trauma and pain since the tissues are more fragile. According to Grahame [(18) p. 485] “Even a single hypermobile joint may suffer any or all of the consequences of laxity, including a tendency to dislocate, develop traumatic synovitis or premature osteoarthritis, or it may just hurt for no visibly obvious reason.” Thus, far from being trivial, the presence of JH should draw attention and lead to a deeper exploration in order to track associated problems such as ligament and tendon problems, joint dislocation/subluxation, chronic arthralgia/myalgia, fatigue, abnormal stature, autonomic, cardiovascular, ocular, neuromuscular, visceral, auditory, and dental pathologies, etc. (19). These should be considered suggestive of an underlying pathology such as a Heritable Disorders of Connective Tissue (HDCTs). Indeed, in this group of disorders, JH is a prominent feature along with fragility of tissues, abnormal skin texture, dysfunctional vessels, and internal organs (20). The HDCTs includes classically Marfan syndrome, Ehlers-Danlos syndromes (EDS), Osteogeneses Imperfecta, and a large list of other genetic disorders, some of them very rare (21). The affected genes encode various connective tissue matrix proteins (collagen, elastin, tenascin, and fibrillin). As consequence, the biochemical structure of fibrous proteins is compromised, altering their physical qualities and resulting in hyperlaxity and mechanical defect (21). In this regard, pain may be present in any HDCTs, but is more prevalent in EDS (19).

With the current specialization and fragmentation of care, patients with ASD and hypermobility-related disorders (HRDs) are seen by different medical fields, such as psychiatry in the case of ASD, and musculoskeletal disciplines and genetics in the case of HRDs. Therefore, a link between these conditions is rarely established in clinical setting despite a scarce but growing body of research suggesting that both conditions co-occur more often than expected by chance (22–24).

This work proposes an overview of the link between ASD and HRDs. We expect to raise awareness among health professionals on the interconnection between these clinical entities, in order to better identify those patients with ASD who may be susceptible to chronic pain.

## Autism, Joint Hypermobility (JH) and Hypermobility-Related Disorders (HRDs)

Current clinical descriptions of young children with autism include hypotonia, joint laxity, clumsiness, apraxia, and toe walking as common findings (25). Interestingly, similar features have been also described in people with HRDs (26–28).

To the best of our knowledge, the first systematic study exploring the association between JH (non-syndromic) and autism according to DSM-IV criteria (1) is that of Shetreat-Klein et al. (29). These authors assessed the range of joint mobility at the elbow, wrist, metacarpo-phalangeal joint, and ankle in children with ASD aged 4 years old in average, and in matched healthy children (*n* = 38 in each group). Results showed that the joints of children with autism were significantly more supple than their typically developing peers. In the same vein, the study of Eccles et al. (30) explored JH and autonomic dysfunction in a group of adult patients with neurodevelopmental disorders (*n* = 205), including patients with autism although the exact number of these subjects was not reported. Results showed that the rate of JH and autonomic symptoms were significantly higher among people with neurodevelopmental disorders than in the control group. More recently, Glans et al. (31) explored the potential association between JH and autistic traits in the general population. One thousand thirty-nine Swedish adults responded to the Five-point questionnaire for JH (32), and others instruments assessing neurodevelopmental traits including the abridged version of the 50-item Autism Spectrum Quotient (33). No link was observed between JH and autistic traits in this study, which lead the authors to suggest that this association is limited to clinical populations only.

Most of the data linking ASD and JH, center around genetic syndromes featuring JH and/or HDCTs. For instance, Fragile X syndrome which is caused by an alteration of the FMR1 gene, is the second cause of intellectual disability among males, and the most frequent genetic comorbidity of ASD (30–50%). In this syndrome, an underlying connective tissue anomaly is presumed since signs such as JH (50%), soft skin, scoliosis, flat feet, and pectus excavatum among others are common in those affected (34).

The Chromosome 2q37 Deletion Syndrome, which is characterized by three major clinical features (developmental delay, intellectual disability, skeletal malformations, and facial dysmorphism) also include in their phenotypical description JH, hypotonia, and dislocations. It has been reported that around 17–50% of patients with this syndrome, also have ASD (35).

Concerning HDCTs, the work by Blair et al. (36) which examined comorbidity among Mendelian and complex diseases by mining the medical records of over 110 million patients, observed significant clinical comorbidities between Marfan syndrome (i.e., HDCTs characterized by marfanoid habitus, aortic aneurysm, and ectopia lentis) and neuropsychiatric conditions such as ASD. These results had not been reported before. Recently, Balasubramanian et al. (37) reported a higher incidence of ASD in people with Osteogeneses Imperfecta (i.e., HDCTs characterized by brittle bones and blue sclerae). Ten out 102 patients in their cohort have ASD while in the general population ASD is estimated of 1 in 100.

Among HDCTs, Ehlers-Danlos syndromes exhibit the greatest clinical overlap with ASD in literature.

## Ehlers-Danlos Syndromes (EDS) and Autism

EDS is not a single disease, but a group of clinically and genetically heterogeneous conditions characterized by JH, skin hyperextensibility and tissue fragility (38). Since collagen is widely distributed through the body, the manifestations of EDS are multi-systemic and often pain-associated.

**Figure 1:**
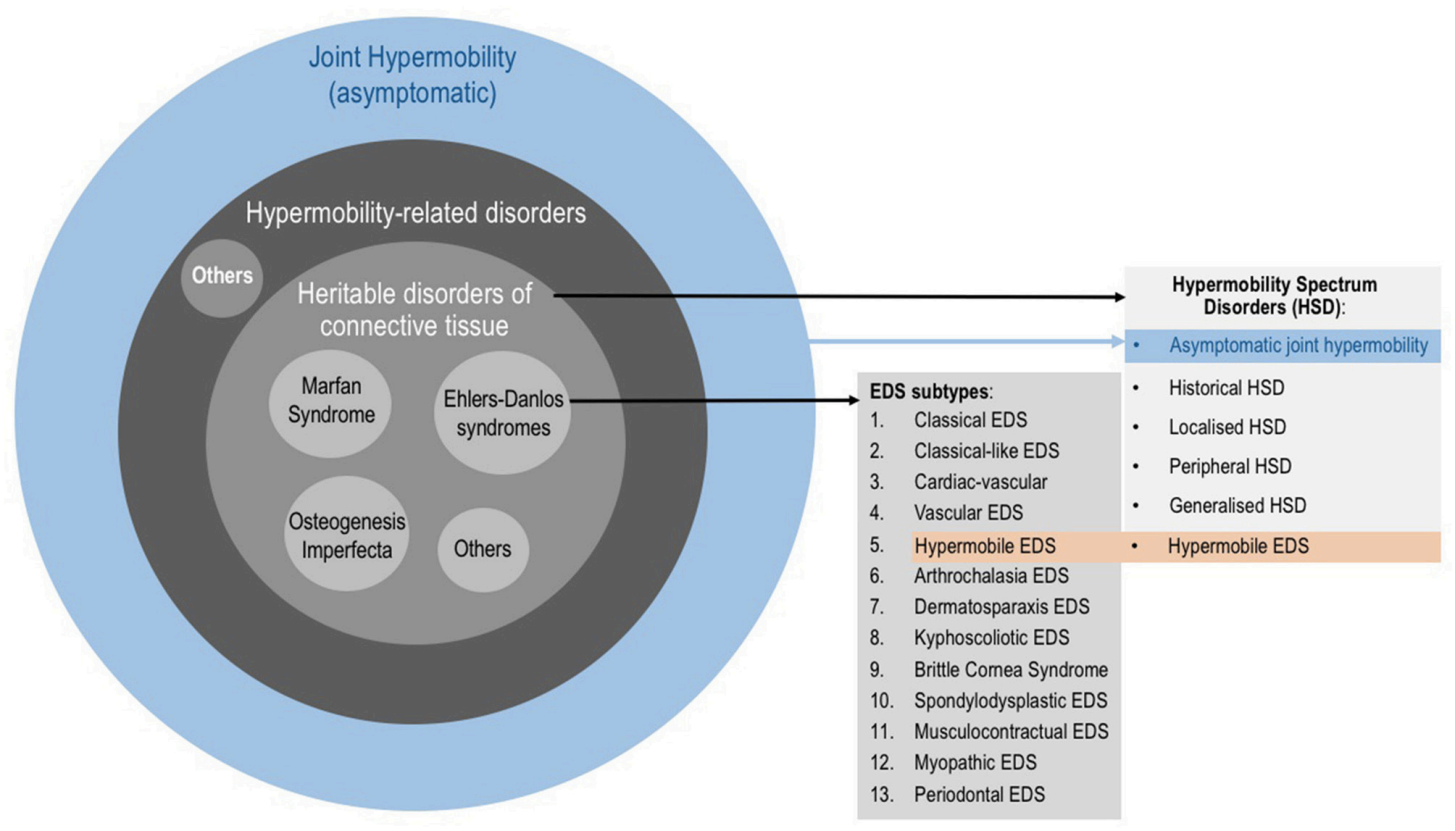
Categories involving joint hypermobility. Joint hypermobility (JH) is frequent in the general population and is not a problem per se. When JH is accompanied by symptoms, it could underlie a hypermobility-related disorder such as a Heritable Disorder of the Connective Tissue (HDCT). HDCT classically include Marfan syndrome, Osteogenesis imperfecta and Ehlers-Danlos syndromes (EDS). The 2017 EDS classification describes 13 subtypes. It also describes the so-called Hypermobility Spectrum Disorders (HSD), which is a group of clinical conditions with symptomatic JH but not fulfilling criteria of any EDS subtype. HSD is understood as a continuum on which JH ranges from asymptomatic JH through to hypermobile EDS (hEDS) as part of the EDS.

The Revised 2017 International Classification for EDS (38) describe 13 subtypes (Figure 1) which range from mild (although debilitating) to life threatening. The prevalence of all EDS is estimated at 1/5,000 (20). The hypermobile subtype (hEDS) is the most common and accounts for 80–90% of EDS cases (39). As in ASD, the genetic background of hEDS has not yet been elucidated, therefore this is the only EDS subtype for which the diagnosis remains clinical. EDS, and especially hEDS are often under-recognized (40). The diagnostic delay was estimated at 14 years for half of EDS patients, and 28 years for a quarter of patients constituting the longest diagnostic delay among 16 rare diseases (41). Recent changes in EDS nosology now indicated that hEDS lies at the end of a spectrum (the so-called Hypermobility Spectrum Disorders; Figure 1) which includes intermediate phenotypes presenting JH plus other symptoms but not fulfilling criteria for an EDS (17). Thus, the Joint Hypermobility Syndrome described by rheumatologists (42) is now part of the hypermobility spectrum.

It is worth mentioning that there remains disagreement amongst experts regarding the new EDS nosology is not an easy issue (43, 44). As in ASD, there is ongoing debate concerning the description of hEDS in particular, as to whether it is a genetic disease or a clinical syndrome is still relevant. In this regard, these new criteria will be revised during 2018 by the International Consortium on EDS.

The first attempt to characterize EDS patients from a psychosocial perspective was the study by Lumley et al. (45). In this study, 44 adults and 7 children with EDS of various subtypes were tested and interviewed. The results in children (aged 7–12-years-old) showed some features compatibles with ASD such as impaired social competence (57 % of the sample), internalizing problems (43%), and aberrant behaviors (29%) although no specific assessment for autism was applied in this study. Moreover, to date eight case reports have been published concerning the potential association between ASD and EDS (22, 46, 47). First, Fehlow and Tennstedt (48) in a German publication presented the case of a 15-year-old boy with an autistic syndrome and EDS type I (current classical type) having JH, skin extensibility, moderate bleeding tendencies and deformity of the thorax. Sieg (49) for his part, described a 13-year-old boy presenting ASD symptoms such as impaired social skills, unusual interests, language delay, mannerisms, lack of awareness of feelings of others, and physical particularities such as JH, Gorlin's sign (i.e., the ability to touch the tip of the nose with the tongue), hyperextensible skin with velvety texture, and a trend to dislocations among others. A diagnosis of EDS type II (current classical type) was confirmed by geneticists. Later, Tantam et al. (50) highlighted the co-occurrence of Asperger's syndrome (now part of ASD) and HDCTs through the presentation of three cases (two girls and one man) with lifelong hyperlaxity and muscular incoordination among others. The authors discarded EDS as a diagnosis due to the absence of skin elasticity in these patients, and concluded a Marfan-like disorder of connective tissue. However, the clinical descriptions are compatible with hEDS (47) in which skin hyperextensibility may be absent. In addition, another case combining high-functioning autistic disorder and EDS was reported by Takei et al. (51). In which a 17-years-old boy presented with highly flexible fingers and toes, JH and skin hyperelasticity. His mother was also diagnosed with EDS, but the subtype in both cases was not mentioned. Similarly, we reported a case of a 12-years-old boy diagnosed with ASD who was referred to rehabilitation medicine due to articular and muscular pain, gait problems and chronic fatigue. The physical exploration revealed JH, history of recurrent sprains and blocks, thin skin with abnormal scarring, easy bruising, cutaneous hyperesthesia, hypotonia, dysautonomia symptoms (excessive sweating, poor thermoregulation, unexplained fever episodes, dry eyes and mouth, dizziness), gastrointestinal problems, severe headaches, and proprioceptive dysfunction (clumsiness, frequent trips and falls, difficulties in gripping and holding objects). The patient's father and brother had similar signs (although milder) and the patient's mother had been diagnosed with fibromyalgia. Finally, a diagnosis of hEDS was obtained for this patient (22). The last case report is that by Cravero et al. (52), who described a 21-years-old man with Cornelia de Lange, Ehlers-Danlos syndrome (classic type), and severe autistic syndrome. Concerning EDS features, he presented JH, pale and hyperextensible skin, abnormal healing with widened anthropic scars, hemorrhagic syndrome, and a family history of EDS.

Recently, Lipsker et al. (11) described the case of a 6-years-old girl with severe chronic pain since very early age (headaches, joint and muscle pain), and comorbid ASD and attention deficit/hyperactivity disorder (ADHD). The authors also describe JH and fatigue in this patient, as well as antecedents of chronic pain in her relatives. After consulting several clinics and trying different treatments, improvements in pain and functioning were obtained with methylphenidate medication and parental behavioral training. Although the possibility of a HDCTs was not evoked, considering the combination of JH, chronic pain and neurodevelopmental disorders, a hypermobility-related disorder such as EDS should be hypothesized (23), as was noted by Fernell and Ronge (53) in a letter to the editor about this case report.

The anecdotal evidence provided by the aforementioned clinical descriptions, which correspond mainly to patients with secondary rather than idiopathic ASD, is supported by the study of Cederlöf et al. (54) in a large cohort of EDS and hypermobility syndrome patients (*N* = 1,771). In this work, EDS and hypermobility syndrome subjects were compared to matched controls in relation to antecedents of psychiatric disorders. Results showed that ASD was overrepresented in EDS patients (2.9% vs. 0.4% in controls; RR 7.4, 95% CI 5.2–10.7). Similarly, ASD was diagnosed in 1.6% of patients with hypermobility syndrome compared to 1.4% in controls (1.6% vs. 1.2%; RR 1.4, 95% CI 1.1–1.6). In addition, more cases of ASD were found in hypermobility syndrome siblings compared to control sibling (ASD in 0.6% vs. 0.5%, respectively; RR 1.3, 95% CI 1.1–1.7).

## Explaining the Link Between Autism and HRDs

The etiological mechanisms underlying the comorbidity between ASD and HRDs are poorly understood. According to Tamtam et al. (50), a disorder of the connective tissue may result in central nervous system abnormalities. This was probably the case of an EDS patient with epilepsy reported by Cupo et al. (55), in which postmortem explorations showed structural brain abnormalities that may be related to the connective tissue disorder. Moreover, Eccles et al. (56), reported structural brain differences between subjects with and without JH in areas involved in emotion processing, attention, cognitive control of pain, and negative emotions (bilateral amygdala, anterior cingulate, parietal lobe), as well as a negative correlation between JH and superior temporal volume, which is an area related to processing social and emotional signals. Differences in amygdala and superior temporal cortex anatomy have been also observed in autism (57).

Brain heterotopias (i.e., neuronal migrational abnormalities) have been reported in ASD (58) and in EDS (55, 59, 60) providing another clue in understanding the overlap between both conditions. In addition, studies in human and animal models indicate immunological dysfunction in ASD (61), while recent research highlights the co-ocurrence of mast cell dysregulation in EDS (62, 63) suggesting problems in the immune system. Moreover, endocrine dysregulation has been postulated as a potential risk factor of ASD [e.g., maternal diabetes, polycystic ovary syndrome, etc.; (64)]. Endocrine involvement has also been identified in EDS. Hugo-Rodin et al. (65) reported a high prevalence of gynecological symptoms in women with hEDS, of which a subgroup was sensitive to hormonal fluctuations with an increase in symptoms severity during puberty, prior to menstruation, during the postpartum period, and on oral contraception. Authors suggested that hormones may play a modulatory effect in hEDS. Recently, the study of Casanova et al. (24) observed that women with ASD and JH reported significantly more immune- and endocrine-mediated conditions than those without JH. These results shed new light on the potential comorbidity between ASD and HRDs.

**Figure 2:**
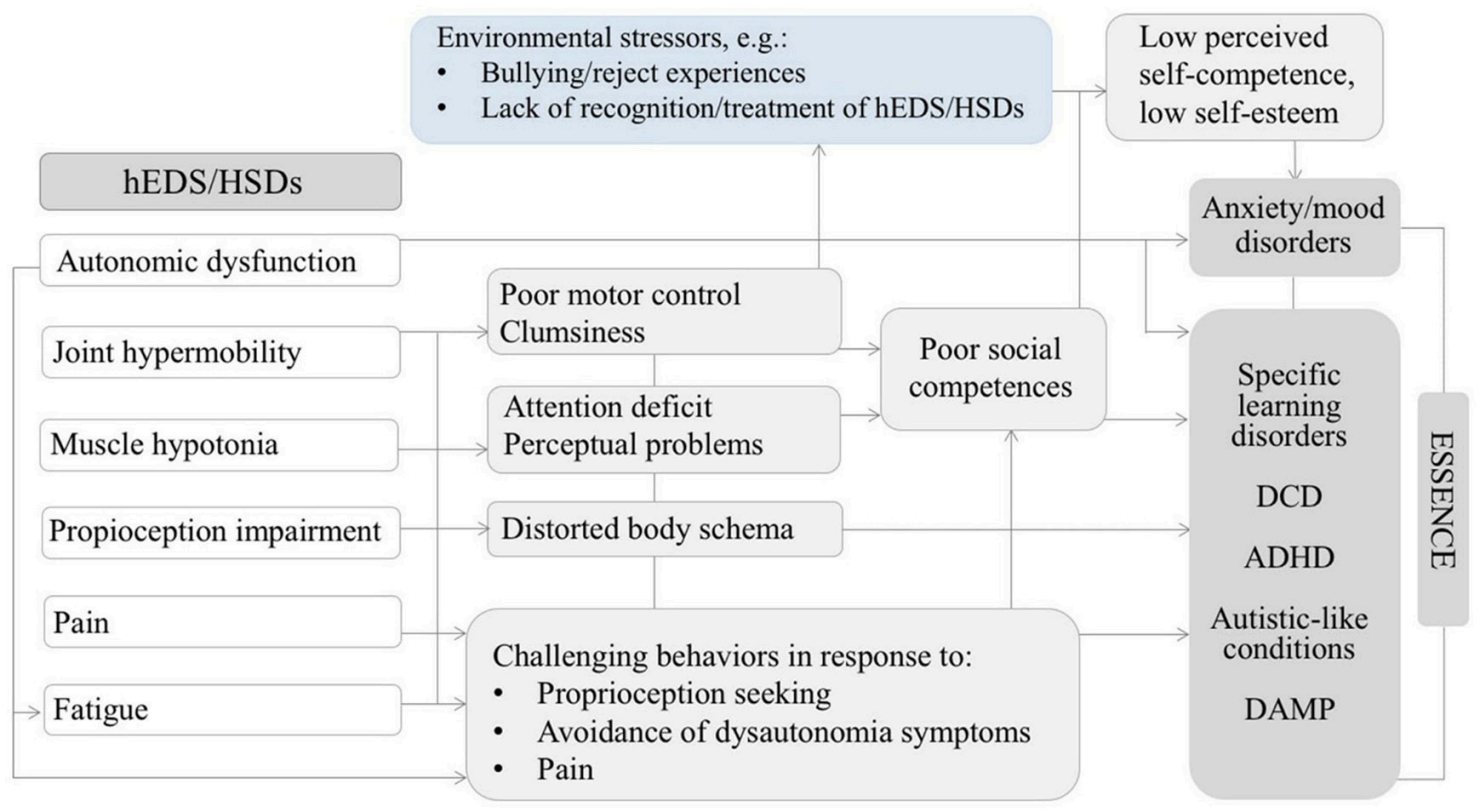
Diagram illustrating possible relationships between some features of hEDS/HSDs might contributing to neurodevelopmental disorders and psychopathology in the developmental age. Adapted by permission from Springer Nature: ADHD Attention Deficit and Hyperactivity Disorders. Attention-deficit/hyperactivity disorder, joint hypermobility-related disorders and pain: expanding body-mind connections to the developmental age, Baeza-Velasco et al. (66) Copyright 2018. hEDS/HSDs: hypermobile Ehlers-Danlos syndrome/hypermobility spectrum disorders. ADHD: attention deficit/hyperactivity disorder. DCD: developmental coordination disorder. DAMP: deficits in attention, motor control and perceptual abilities (67). ESSENCE: early symptomatic syndromes eliciting neurodevelopmental clinical examinations (68).

Tamtam et al. (50) also evoked the hypothesis of an indirect link between ASD and HRDs. This raises the question whether connective tissue abnormalities alter motor development and proprioception preventing the optimal acquisition of non-verbal communication skills, which may lead to autistic traits such as impairments in social interactions. The Figure 2 illustrate this idea showing possible connections between HRDs (specially hEDS and hypermobility spectrum disorders), and neurodevelopmental outcomes including autistics traits (66).

Finally, ASD and HRDs may be pleiotropic manifestations of a common genetic milieu that deserve to be better scrutinized.

## Autism, HRDs and Pain

As has been suggested, a link between ASD and HRDs (specially EDS) theoretically implies a susceptibility to a wide range of pain (mainly musculoskeletal but also headaches and visceral pain). In this sense, there is evidence of higher rates of pain symptoms in EDS patients when there is comorbidity with a psychiatric disorder (69). In addition, mast cell activation syndrome, which has been associated to several painful conditions [e.g., migraine, atopic dermatitis, pelvis and bladder pain, inflammatory bowel pain, fibromyalgia, vulvodynia, self-injurious behaviors associated pain, etc. (70)] is frequent in ASD (71) and EDS (62).

Despite these suggestive data, confirmation by systematic studies about the presence of pain in ASD with HRDs is needed. In this sense, Casanova et al. (24) contributed some of the first data. Through a survey via internet, this group explored adult women on the autism spectrum with and without JH (*n* = 85 vs. *n* = 20, respectively) and estimated the prevalence of immune and endocrine mediated conditions. It was observed that the hypermobile ASD group presented significantly higher rates of autoimmune disorders (45% vs. 13%; *p* = 0.02), but also pain-associated endocrine symptomatology such as dysmenorrhea and endometriosis (85% vs. 28%; *p* < 0.001 and 30% vs. 5%; *p* = 0.01 respectively) compared to the non-hypermobile ASD group. In addition, all participants in the hypermobile ASD group suffered arthralgia, and 75% from other types of chronic pain (including fibromyalgia) compared to 29 and 31%, respectively, in the non-hypermobile ASD group (*p* < 0.001). These results concern only females and a subpopulation capable of answering online self-questionnaires, for which authors assume an IQ > 70. In this regard, studies in males and individuals with autism and intellectual disability are needed in order to extend explorations to a more representative ASD population.

## Conclusion

ASD and HRDs, specially hEDS, are conditions with a strong genetic component, a polymorphic clinical presentation, appearing both in infancy, and sharing several phenotypical features (35). Although existing data does not allow to ascertain increase prevalence of ASD in HRDs, as well as shared underlying patho-mechanisms between both conditions, there is increasing evidence suggesting that these co-occur more often than expected by chance. This requires be confirmed by further investigation which should consider the recent nosological changes both in EDS and the hypermobility spectrum disorders [see (17, 38)], and in ASD (72).

Disseminate knowledge about this potential connection can be highly useful in clinical context since it allows the clinician the awareness of potential pain-related symptoms in a population in which it is extremely challenging to screen for and manage pain. Beyond the methodological barriers to explore pain in people with ASD, and as (69) stated, mental-health related stigma can prevent more depth investigations into an underlying cause of systemic complaints, or to the exacerbation of behavioral problems and/or comorbid psychopathology in the case of ASD patients, delaying the recognition of HRDs. Conversely, patients primarily treated for painful conditions related to hypermobility, should be screened for neurodevelopmental abnormalities. A broad image of each patient, including somatic and psychological aspects, will help to ensuring proper care.

Moreover, once the association between autism, HRDs and pain have been established, it is necessary to consider antalgic strategies targeted to this specific subgroup of patients. Such therapeutic strategies are unexplored so far, hence clinicians are underprepared to manage complex clinical pictures. Thus, future avenues for research that deserves more attention includes the confirmation of comorbidity between ASD and HRDs, the elucidation of its etiology and clinical significance, and the most appropriate management approach for these cases.

## Author Contributions

All authors listed have made a substantial, direct and intellectual contribution to the work, and approved it for publication.

### Conflict of Interest Statement

The authors declare that the research was conducted in the absence of any commercial or financial relationships that could be construed as a potential conflict of interest.
